# Chemical Composition and Antimicrobial Activity of Essential Oils

**DOI:** 10.3390/plants12040800

**Published:** 2023-02-10

**Authors:** Stefania Garzoli

**Affiliations:** Department of Drug Chemistry and Technology, Sapienza University, 00185 Rome, Italy; stefania.garzoli@uniroma1.it

This Special Issue entitled “Chemical Composition and Antimicrobial Activity of Essential Oils” focuses on the chemical characterization of essential oils (EOs) through analytical techniques that are necessary for the identification and quantification of individual compounds. It is also centered on the evaluation of their antimicrobial activities through biological assays performed to test their effects.

In general, the composition of EOs is rich and complex and consists of compounds belonging to various chemical classes; therefore, it is necessary to resort to highly sensitive and scientifically valid investigation methods. In this way, it is possible to describe the chemical profiles of EOs, which is fundamental for achieving a more in-depth understanding of their secondary metabolites and their potential effects; this can also be achieved through composition–activity correlation studies.

This SI collects 12 articles and one review that address different issues but with the same common thread, namely that of reporting the most recent developments in the field regarding the uses and applications of EOs. Specifically, the papers chosen for this editorial are fully consistent with the focus of the SI.

An application of EOs in the food sector has been reported by Al-Askar and colleagues [1]. Penicillium mold was isolated and identified as a new deposition pathogen on Jerusalem artichoke (JA) tubers which are prone to spoilage by mold, as it destroys essential tuber components. A cheap procedure involving EOs was conducted to preserve the nutritional value of the tubers under JA storage conditions. The EOs of cumin and clove, at a concentration of 2%, were selected on the basis on their strong antifungal action. Tubers treated individually with both oils showed less serious effects due to blue mold. Cumin showed superior effects, but the results confirmed the suitable use of both oils as economic and eco-safe products for preserving JA tubers at room temperature to maintain their nutritional value.

Still in the food sector, *Thymus serpyllum* EO, after being chemically investigated to describe its composition (thymol, 18.8%; carvacrol, 17.4%; o-cymene, 15.4%; and geraniol, 10.7% were the main components), was tested for its antimicrobial effects against biofilm-forming bacteria. In particular, its inhibitory effect was observed on the biofilm of *Bacillus subtilis* and on that of *Penicillium crustosum*, where the vapor phase of the oil proved to be particularly effective. Effects on biofilm were detected using the MALDI-TOF MS Biotyper. The findings demonstrated that *T. serpyllum* EO in the vapor phase can be used for the preservation of root vegetables and as an inhibitor of the growth of *Penicillium* on bread [2].

In another study, the possibility of using mixtures of EOs and the respective hydrosols (HYs) of *Origanum vulgare*, *Thymus vulgaris*, *Citrus limon*, and *Citrus sinensis*, obtained via hydrodistillation, to control the gray mold disease caused by *Botrytis cinerea* Pers ex. Fr., was evaluated.

Encouraging results of antifungal activity in vitro were obtained for both thyme and oregano EOs. The activity was mainly due to the volatile compounds thymol and carvacrol contained in the two oils. The damage on *B. cinerea* tomatoes (in vivo test) was significantly reduced by the application of a thyme EO/HY blend, thus demonstrating that the application of the two-component blend improved antifungal activity. In all experiments, the treatments tested were more effective than using BC-1000^®^ Dust, which is a non-toxic fungicide derived from grapefruit seeds (*Citrus paridisi*) and pulp extracts. Therefore, EO/HYS blends could be developed as potential formulations for in vivo application in agricultural disease control, thus avoiding the use of synthetic fungicides [3].

A comparative study in terms of chemical composition and cytotoxic and antimicrobial activity was conducted on twenty-seven samples of pepper fruits (CF). The number of compounds identified that belong to different chemical classes was 71, with a large percentage variation between the different compounds. The results of the cytotoxicity tests showed that the percentage of inhibition was in the range of 9–47 (MCF7) and 4–41 (HCT116), while the zone of inhibition (mm) for the antimicrobial activity was found to be 0.0–17 (*P. aeruginosa*) > 0.0–13 (*E. coli* and *S. aureus*). 

Moreover, samples with the largest zones of inhibition found via the agar diffusion method (C16, C19, and C26) presented minimum MIC and MBC values after further evaluation compared to *P. aeruginosa*, with an MIC and MBC (µg/mL) of 6.3 and 12.5, respectively. The data obtained from a supporting statistical analysis confirmed that the sample C16 had the best activity, followed by C5 (green long serrano, Holland), C13 (green long chili, Saudi—the best for being cytotoxic), C19 (red small chili, Saudi), and C26 (orange small baby pepper, Spain—the best antimicrobial) [4].

In general, EOs obtained from wild Mediterranean plants are rich in bioactive volatile substances and, for some of them, it has also been demonstrated that they are effective against the pathogens *Aphanomyces astaci*, the causative agent of crayfish plague, and *Saprolegnia parasitica*, the causative agent of saprolegniosis in fish.

These pathogens are the cause of serious economic losses in aquaculture and, because of this, efforts were made to find an inhibitory agent that was of natural origin and was basically non-toxic. So, the EOs of sage, rosemary, and laurel were tested for this purpose. The GC analyses revealed a profile rich in monoterpenes and the results of the tests showed that the mycelium and zoospores of *A. astaci* were more sensitive than those of *S. parasitica*, where only sage EO completely inhibited the mycelium growth. The observed inhibition could be related to the presence of camphor as the most abundant component, but without excluding a synergistic effect between several compounds. These results open a perspective for the environmentally friendly and sustainable control of oomycete diseases in salmonid and shrimp aquaculture through the application of essential oils [5].

In general, the chemical composition can indicate a high level of biodiversity among species. With this in mind, some studies were conducted on different populations of thyme collected in five locations in western Romania. GC-MS analyses identified 60 chemical compounds belonging to three chemotypes: thymol (three populations), geraniol (one population), and carvacrol (one population). *Thyme vulgaris* L. is distinguished by a high content of thymol, while species of spontaneous flora (*Th. Odoratissimus* and *Th. pulegioides*) contain, in addition to thymol, appreciable quantities of carvacrol and geraniol.

For all EOs, their antimicrobial activity on *Staphylococcus aureus*, *Streptococcus pyogenes*, *Esherichia coli*, *Pseudomonas aeruginosa*, *Shigella flexneri*, *Salmonella typhimurium*, *Haemophilus* influenzae type B, *Candida albicans*, and *Candida parapsilopsis* was tested. The EOs showed activity against Gram-positive, Gram-negative, and fungal pathogens, and the most sensitive strains were *S. pyogenes*, *S. flexneri*, *S. typhimurium*, and *C. parapsilopsis*. *T. odoratissimus* had a higher positive inhibition rate than the other EOs studied, regardless of the administered oil concentration. A correlation between antimicrobial activity was also evaluated for Thymus EOs and the presence of phenolic compounds, such as thymol and carvacrol. So, these oils could be used in the prevention of bacterial or fungal infections in plants, animals, or humans, being 100% natural and non-toxic in small quantities [6].

The EOs of the aerial parts (A) and flowers (F) of *Rosa banksiae* var. *banksiae* Ait. (RBW), *Rosa polyantha* Thunb. “orange fairy” (RPO), and *Rosa polyantha* Thunb. “white fairy” (RPW), family Rosaceae, were investigated via GC-MS, and their antimicrobial activity against four bacteria and four fungi was evaluated. The antimicrobial activity was investigated using the well diffusion method. Two hundred and fifty-three compounds were identified and hydrocarbon terpenoids and oxygenated terpenoids were the predominant classes identified. Among these, the major components in RBW-A, RPO-A, and RPW-A were n-undecane (14.40, 19.36, and 9.21%), n-dodecane (14.54, 22.13, and 8.39%), and yomogi alcohol (8.41, 10.53, and 6.28%), respectively, whereas RBW-F, RPO-F, and RPW-F contained n-heptadecane (16.70%), n-undecane (7.98%), and β-phellandrene (22.78%), respectively. The tested EOs showed moderate antifungal activity against *Aspergillus fumigatus* compared to amphotericin B, while weak activity or no activity were observed against some Gram-positive and Gram-negative bacteria [7].

Dermatophyte infections represent an important public health concern. *Trichophyton rubrum* and *T. mentagrophytes* are the predominant dermatophytes in cutaneous infections. Although terbinafine represents the preferred treatment, its clinical use is hampered by side effects, drug–drug interactions, and the emergence of resistant clinical isolates. 

Selected Apiaceae EOs (ajowan, coriander, caraway, and anise) were screened to improve the antifungal activity of terbinafine against *T. rubrum* and *T. mentagrophytes*. The chemical profiles of EOs were analyzed via GC and the minimal inhibitory concentration (MIC) and minimal fungicidal concentration (MFC) of EOs/main compounds were determined. Furthermore, the checkerboard microtiter method was used to identify putative synergistic combinations of EOs/main constituents with terbinafine. The binary associations of tested EOs with terbinafine were found to be synergistic against *T. rubrum*, with FICI values of 0.26–0.31. At the tested concentrations (6.25–25 mg/L), EOs did not exert cytotoxic effects towards human neutrophils. Anise EO was the most potent inhibitor of IL-1b release, while coriander EO displayed the highest inhibition on IL-8 and TNF-a production. Therefore, the use of such mixtures might provide a better outcome than monotherapy in terms of side effects and toxicity, but also in decreasing the emergence of resistant strains.

In conclusion, the synergistic combinations of terbinafine and investigated Apiaceae EOs could be a starting point in the development of novel topical therapies against *T. rubrum*-related dermatophytosis [8]. 

The liquid and vapor phase chemical compositions of *Laurus nobilis*, *Salvia officinalis*, and *Salvia sclarea* EOs and HYs were investigated to define their liquid and volatile chemical compositions, using GC/MS and HS-GC/MS techniques. 1,8-Cineole (42.2%, 33.5%) and α-pinene (16.7%, 39.0%) were the main compounds of *L. nobilis* EO; 1,8-cineole (30.3%, 48.4%) and camphor (17.1%, 8.7%) were the main compounds of *S. officinalis* EO; and linalyl acetate (62.6%, 30.1%) and linalool (11.1%, 28.9%) were the main compounds of *S. sclarea* EO for the liquid and vapor phases, respectively. The chemical profiles of HYs were characterized by 1,8-cineole (65.1%, 61.4%) as a main constituent of *L. nobilis* and *S. officinalis* HYs, while linalool (89.5%) was the main constituent of *S. sclarea* HY. The antioxidant activity and antimicrobial properties were also investigated against five different bacterial strains, such as *Escherichia coli*, *Pseudomonas fluorescens*, and *Acinetobacter bohemicus* among Gram-negative bacteria and *Bacillus cereus* and *Kocuria marina* among Gram-positive bacteria. *L. nobilis* and *S. officinalis* EOs demonstrated considerable antibacterial activity, while *S. sclarea* EO proved to be less effective. Remarkably high antioxidant activity was determined for *L. nobilis* and for *S. sclarea*. The HYs instead showed extremely low antioxidant activity [9].

The chemical composition via GC-MS and the antioxidant, antifungal, and insecticidal activities of EO extracted from Moroccan lavender (*Lavandula dentata*) were investigated. The major component was linalool (45.06%), followed by camphor (15.62%) and borneol (8.28%).

Antifungal activity was assessed by calculating the inhibition of growth of *Alternaria alternata*, *Botrytis cinerea*, and *Fusarium oxysporum*, and the repellent potential and toxicity of EOs were investigated against *Callosobruchus maculatus*. The EOs showed significant antioxidant activity and an inhibitory effect on mycelial growth against tested fungi. Furthermore, the EOs caused total mortality of adult *C. maculatus*. A significant reduction in numbers of eggs laid (99.2%) and emergence (100%) was observed in a dose-dependent manner. So, based on the collected data, the EO *Lavandula dentata* could be an environmentally friendly alternative bio-fungicide and bio-insecticide [10].

The chemical composition of *Brocchia cinerea* (Delile) Vis. EO using GC-MS and the antimicrobial properties in terms of insecticidal and repellent effectiveness against *Callosobruchus maculatus* were evaluated. Thujone (24.9%), lyratyl acetate (24.32%), camphor (13.55%), and 1,8-cineole (10.81%) were the most prominent compounds. For the antimicrobial assay, the yeast *Candida albicans* was very sensitive to the EO, followed by *Staphylococcus aureus*, while *Fusarium oxysporum* was the mycelia strain extremely sensitive to the utilized EO compared to the two species of *Aspergillus* (*A. flavus* and *A. niger*). The results obtained using the microdilution method also showed that *Pseudomonas aeruginosa* was very sensitive to the EO.

Based on the obtained results, *B. cinerea* EO could be used as a biopesticide in the integrated management of *C. maculatus*, and it could also be used as an antimicrobial alternative to conventional antibiotics in the control of resistant strains (fungi and bacteria) [11].

Severe Acute Respiratory Syndrome Coronavirus 2 (SARS-CoV2), the causative agent of the coronavirus disease 2019 (COVID-19), has seriously threatened global health. 

Several EOs provide a rich source of compounds with valuable antiviral activities. *Agathus robusta* EO bark was investigated for its chemical composition and its antiviral activity against SARS-CoV2. Overall, 26 constituents were identified via GC-MS analysis.

α-Pinene, tricyclene, α-terpineol, limonene, D-camphene, trans-pinocarveol, α-phellandren-8-ol, L-β-pinene, and borneol were the major components. In silico docking of these constituents against viral key enzymes, the spike receptor-binding domain (RBD), main protease (Mpro), and RNA-dependent RNA polymerase (RdRp), using Molecular Operating Environment (MOE) software, revealed good binding affinities of the components to the active site of the selected targets, especially the RBD. In vitro antiviral MTT and cytopathic effect inhibition assays demonstrated a promising antiSARS-CoV2 for *A. robusta* bark EO, with a significant selectivity index of 17.5 [12].

Melaleuca is one of the genera of the Myrtaceae family, enriched in tea tree oil (TTO). Tea tree oils of *Melaleuca bracteata* and *Melaleuca alternifolia* are of prime importance and have antioxidant and antimicrobial properties. Terpinen-4-ol and 1,8-cineole are major constituents of *M. alternifolia* oil.

Tea tree oil has gained much fame and become an integral part of the pharmaceutical, agriculture, food, and cosmetic industries of the world. Its importance lies in its complex composition which is characterized by high levels of antimicrobial and antioxidant compounds such as terpinen-4-ol, methyl eugenol, and 1,8-cineole. The review paper provides comprehensive information regarding the antioxidant and antimicrobial activities of tea tree oil and its potential applications in agriculture [13].
